# Matrix metalloproteinase-10: a novel biomarker for idiopathic pulmonary fibrosis

**DOI:** 10.1186/s12931-015-0280-9

**Published:** 2015-09-29

**Authors:** Akihiko Sokai, Tomohiro Handa, Kiminobu Tanizawa, Toru Oga, Kazuko Uno, Tatsuaki Tsuruyama, Takeshi Kubo, Kohei Ikezoe, Yoshinari Nakatsuka, Kazuya Tanimura, Shigeo Muro, Toyohiro Hirai, Sonoko Nagai, Kazuo Chin, Michiaki Mishima

**Affiliations:** Department of Respiratory Medicine, Graduate School of Medicine, Kyoto University, 54 Shogoin-Kawaharacho, Sakyo-ku, Kyoto 606-8507 Japan; Department of Respiratory Care and Sleep Control Medicine, Graduate School of Medicine, Kyoto University, 54 Shogoin-Kawaharacho, Sakyo-ku, Kyoto 606-8507 Japan; Louis Pasteur Center for Medical Research, Kyoto, Japan; Department of Diagnostic Pathology, Graduate School of Medicine, Kyoto University, 54 Shogoin-Kawaharacho, Sakyo-ku, Kyoto 606-8507 Japan; Department of Diagnostic Imaging and Nuclear Medicine, Graduate School of Medicine, Kyoto University, 54 Shogoin-Kawaharacho, Sakyo-ku, Kyoto 606-8507 Japan; Kyoto Central Clinic/Clinical Research Center, Sakyo-ku, Kyoto Japan

**Keywords:** Matrix metalloproteinase-10, Idiopathic pulmonary fibrosis, Biomarker

## Abstract

**Background:**

Matrix metalloproteinases (MMPs) are believed to be involved in the pathogenesis of idiopathic pulmonary fibrosis (IPF), and MMP-7 has been described as a useful biomarker for IPF. However, little is known regarding the significance of MMP-10 as a biomarker for IPF.

**Methods:**

This observational cohort study included 57 patients with IPF. Serum MMPs were comprehensively measured in all patients, and the relationships between these markers and both disease severity and prognosis were evaluated. Bronchoalveolar lavage fluid (BALF) MMP-7 and -10 levels were measured in 19 patients to investigate the correlation between these markers and their corresponding serum values. Immunohistochemical staining for MMP-10 was also performed in IPF lung tissue.

**Results:**

Serum MMP-7 and -10 levels correlated significantly with both the percentage of predicted forced vital capacity (ρ = −0.31, *p* = 0.02 and ρ = −0.34, *p* < 0.01, respectively) and the percentage of predicted diffusing capacity of the lung for carbon monoxide (ρ = −0.32, *p* = 0.02 and ρ = −0.43, *p* < 0.01, respectively). BALF MMP-7 and -10 levels correlated with their corresponding serum concentrations. Only serum MMP-10 predicted clinical deterioration within 6 months and overall survival. In IPF lungs, the expression of MMP-10 was enhanced and localized to the alveolar epithelial cells, macrophages, and peripheral bronchiolar epithelial cells.

**Conclusions:**

MMP-10 may be a novel biomarker reflecting both disease severity and prognosis in patients with IPF.

**Electronic supplementary material:**

The online version of this article (doi:10.1186/s12931-015-0280-9) contains supplementary material, which is available to authorized users.

## Background

Idiopathic pulmonary fibrosis (IPF) is a progressive fibrotic pulmonary disease with a median survival of 3 to 5 years [1]. The clinical course of IPF is variable and unpredictable [1–3]. Because survival prediction requires both a large population and a long follow-up period, surrogate endpoints, such as declines in forced vital capacity (FVC) and diffusing capacity of the lung for carbon monoxide (DL_CO_), are often used as primary endpoints in clinical studies instead of survival [4, 5]. Regarding serum biomarkers, none have been established as a widespread clinical marker for IPF [6, 7]. However, previous studies have suggested that biomarkers, such as Krebs von den lungen-6 [8], surfactant protein-A (SP-A) [9], CC chemokine ligand 18 [10], and matrix metalloproteinase (MMP)-7 [11], may serve as prognostic biomarkers for IPF.

MMPs have also been evaluated as biomarkers for IPF. MMPs are a family of proteinases whose active sites contain zinc. MMPs play a major role in the degradation and remodeling of the extracellular matrix (ECM), which consists primarily of glycoproteins, including collagens, proteoglycans, and fibronectin. MMPs also regulate multiple functions, such as cell proliferation, adhesion, migration, differentiation, and apoptosis, and play a pivotal role in the pathogenesis of IPF [12]. It has been reported that MMP-1, -2, and -7 are elevated in the serum and that MMP-3, -8, and -9 are elevated in the bronchoalveolar lavage fluid (BALF) in patients with IPF [2, 13, 14]. MMP-7 is one of the most extensively investigated MMPs in IPF. MMP-1 and -7 may be diagnostic biomarkers that distinguish IPF from other chronic lung diseases such as chronic obstructive pulmonary disease and sarcoidosis [15]. Previous studies have also demonstrated that serum MMP-7 correlates with both FVC and DL_CO_ and is associated with survival in patients with IPF [11, 15], suggesting that MMP-7 may be a useful biomarker. Recently, however, it has been reported that not only MMP-7 but also other MMPs may play a significant role in the pathogenesis of IPF [13, 16].

As is the case with other MMPs, MMP-10 also plays a significant role in the degradation and remodeling of the ECM during tissue repair and vascular remodeling [17, 18], which suggests that MMP-10 is also an IPF biomarker candidate. However, in previous studies that screened serum MMPs in IPF, MMP-10 was not included in the analyses [11, 15]. Therefore, little is known regarding the role of MMP-10 in IPF. In the present study, serum MMPs, including MMP-10, were comprehensively measured in patients with IPF, and their relationships with disease severity, short-term deterioration of pulmonary function, and overall survival were investigated.

## Methods

### Study population

This was an observational cohort study that enrolled 57 consecutive patients with IPF who visited the Department of Respiratory Medicine at Kyoto University Hospital. The diagnosis of IPF was made according to the diagnostic criteria for IPF [1]. Patients who had active malignant disease or whose forced expiratory volume in one second (FEV_1_)/FVC was < 70 % were excluded. The day of serum sampling was set as the baseline, and the patients were prospectively evaluated for clinical deterioration every 6 months from the baseline. Fifteen age- and sex-matched healthy volunteers without lung disease were recruited as healthy controls. Twenty patients with chronic obstructive pulmonary disease (COPD) were also recruited as disease controls. A diagnosis of COPD was made according to the Global Initiative for Chronic Obstructive Lung Disease (GOLD) criteria [19]. The present study was approved by the Ethics Committee of Kyoto University, and informed consent was obtained from all patients.

### Physiological measurements and bronchoalveolar lavage

The pulmonary function test (PFT) [20], the 6-minute walk test (6MWT) [21], and the arterial blood gas (ABG) while breathing room air and bronchoalveolar lavage (BAL) [22, 23] were performed according to published guidelines.

### MMP analysis

Serum and BALF samples were centrifuged immediately following sampling, and the supernatants were stored at -80 °C until needed for analysis. Serum MMP-1,-2, -3, -7, -8, -9, -10, -12, and -13 and BALF MMP-7 and -10 were analyzed using the Bio-Plex Pro Human MMP Panel (Bio-Rad Laboratories, Hercules, CA, USA). Serum samples were quantified according to the manufacturer’s instructions using the Bio-Plex 200, a multiplex cytokine array system. BALF samples and their standards were diluted using bovine serum albumin-phosphate-buffered saline.

### Outcome evaluations

The outcome evaluations included clinical deterioration within 6 months from baseline and overall survival. Clinical deterioration was defined as a composite outcome consisting of admission due to respiratory failure, death, ≥ 10 % decline in the percentage of predicted FVC (%FVC), or ≥ 15 % decline in the percentage of predicted DL_CO_ (%DL_CO_)_._ We analyzed whether MMPs were associated with clinical deterioration and overall survival in patients with IPF.

### MMP-10 immunostaining

Lung tissue specimens obtained from 4 patients with IPF were used for immunohistochemical staining, which was performed on 5-μm paraffin-embedded sections of lung tissue to identify MMP-10-expressing sites using the standard streptavidin-biotin-peroxidase complex method. For antigen retrieval, slides were immersed in citrate buffer and heated in a microwave oven. A rabbit anti-human MMP-10 antibody (Abcam, Cambridge, MA, USA) was applied as a primary monoclonal antibody (1:500 dilution). Positive staining was visualized using 3,3’-Diaminobenzidine. A pathologist evaluated the localization of MMP-10 expression in IPF lung tissue and compared the results with control lung specimens, which were obtained from normal areas distant from the lesions caused by surgically diagnosed organizing pneumonia (OP).

### Statistical analyses

Statistical analyses were performed using JMP 10.0 (SAS Institute Inc., Cary, NC, USA). Comparisons were performed using Fisher’s test, the Mann-Whitney *U* test, or the Steel-Dwass test, where appropriate. Correlations between variables were evaluated using Spearman’s rank correlation coefficient. A logistic analysis was used to predict clinical deterioration within 6 months. To identify the factors predictive of mortality, we used a Cox proportional hazards model. A receiver operating characteristic (ROC) analysis of serum MMP-10 was performed to determine the threshold for predicting clinical deterioration. All analyses were considered statistically significant when *p* < 0.05.

## Results

### Patient characteristics

Fifty-seven patients with IPF were recruited; all patient characteristics are shown in Table 1. Ten (17.5 %) patients were diagnosed by histological confirmation of the usual interstitial pneumonia (UIP) pattern, whereas the other patients were diagnosed based on a definite UIP pattern on high-resolution computed tomography. The mean age of the patients with IPF was 69.4 years, and 51 (89.5 %) patients were male, whereas the mean age of patients with COPD or healthy controls was 70.8 and 65.9 years, respectively. All but one patient with COPD (95.0 %) and all healthy controls were male. No significant difference was observed in age and gender ratios between IPF and COPD nor between IPF and healthy controls (Table 1). All patients with COPD and seven control subjects (46.7 %) had current or former history of smoking. Twenty-five (43.9 %) patients underwent BAL. Three patients had a history of malignant disease: 1 patient had breast cancer, 1 patient had prostate cancer, and another patient had colorectal cancer. No patients suffered disease recurrence over the more than 3 years following their last treatments. Thirteen patients were treated at baseline with prednisolone, an immunosuppressant, or pirfenidone (Additional file 1: Table S1).

**Table 1:** Patient characteristics

	IPF	COPD	Control	*p*-value	*p*-value	*p*-value
	(*n* = 57)	(*n* = 20)	(*n* = 15)	IPF vs. Control	IPF vs. COPD	COPD vs. Control
Age, years	69.4 ± 8.5	70.8 ± 7.1	65.9 ± 7.9	NS	NS	NS
Gender, male	51 (89.5)	19 (95.0)	15 (100)	NS	NS	NS
Current or former smoker	53 (93.0)	20 (100)	7 (46.7)	<0.001	NS	<0.001
Patients who underwent BAL	25 (43.9)	–	–			
Patients who underwent SLB	10 (17.5)	–	–			
FVC, % predicted	84.2 ± 21.3	93.5 ± 20.1	–		NS	
DL_CO,_ % predicted	43.7 ± 14.2	56.6 ± 17.5	–		0.004	
6MWD, m	454 ± 108	–	–			
Minimum SpO_2_ during 6MWT, %	85.7 ± 8.4	–	–			
PaO_2_ at room air, Torr	82.6 ± 13.1	–	–			

The data are presented either as the number (%) or mean ± standard deviation

*IPF* idiopathic pulmonary fibrosis, *COPD* chronic obstructive pulmonary disease, *NS* not significant, *BAL* bronchoalveolar lavage, *BALF* bronchoalveolar lavage fluid, *SLB* surgical lung biopsy, *FVC* forced vital capacity, *DL*_*CO*_ diffusing capacity of the lung for carbon monoxide, *6MWD* 6-minute walk distance, *SpO*_*2*_ oxygen saturation, *6MWT* 6-minute walk test, *PaO*_*2*_ partial pressure of oxygen

### Serum and BALF MMPs in IPF

The serum and BALF concentrations are presented in Table 2. MMP-13 was excluded from the analysis because its mean concentration was under the limit of determination in both patient groups and controls. Serum MMP-1 (*p* < 0.01), MMP-2 (*p* = 0.03), MMP-7 (*p* < 0.01), MMP-8 (*p* = 0.01), MMP-10 (*p* < 0.01), and MMP-12 (*p* < 0.01) concentrations were significantly elevated in patients with IPF compared with controls, whereas serum MMP-2 (*p* < 0.01), MMP-7 (*p* < 0.01), MMP-9 (*p* = 0.03), MMP-10 (*p* < 0.01), MMP-12 (*p* = 0.01) concentrations were significantly elevated in patients with IPF compared with COPD patients.

**Table 2:** Serum and BALF MMP concentrations

	IPF	COPD	Control	*p*-value	*p*-value	*p*-value
	(*n* = 57)	(*n* = 20)	(*n* = 15)	IPF vs. Control	IPF vs. COPD	COPD vs. Control
Serum						
MMP-1, ng/mL	1.52 ± 1.36	0.95 ± 0.74	0.60 ± 0.58	0.005	NS	NS
MMP-2, ng/mL	73.5 ± 60.8	27.6 ± 11.8	41.3 ± 40.6	0.034	<0.001	NS
MMP-3, ng/mL	8.43 ± 10.51	4.54 ± 2.26	3.78 ± 3.00	NS	NS	NS
MMP-7, ng/mL	8.39 ± 4.32	1.72 ± 0.65	2.14 ± 1.41	<0.001	<0.001	NS
MMP-8, ng/mL	1.43 ± 1.50	0.80 ± 0.62	0.47 ± 0.51	0.012	NS	NS
MMP-9, ng/mL	38.7 ± 26.8	23.3 ± 15.7	25.9 ± 24.0	NS	0.030	NS
MMP-10, ng/mL	1.18 ± 0.83	0.16 ± 0.18	0.18 ± 0.18	<0.001	<0.001	NS
MMP-12, ng/mL	0.31 ± 0.23	0.17 ± 0.11	<0.1^a^	<0.001	0.013	<0.001
MMP-13, ng/mL	<0.1^a^	<0.1^a^	<0.1^a^			
KL-6, U/mL	1084.2 ± 724.4	–	–			
SP-D, ng/mL	300.4 ± 223.7	–	–			
BALF						
MMP-7, ng/mL	9.6 ± 10.7	–	–			
MMP-10, ng/mL	0.10 ± 0.09	–	–			

The data are presented as the mean ± standard deviation

*IPF* idiopathic pulmonary fibrosis, *COPD* chronic obstructive pulmonary disease, *MMP* matrix metalloproteinase, *NS* not significant, *KL-6* Krebs von den lungen-6, *SP-D* surfactant protein-D, *BALF* bronchoalveolar lavage fluid

^a^The mean is under the limit of determination

### Correlation between serum MMPs and disease severity

The correlation between pulmonary function and MMPs is shown in Table 3. There were significant correlations between MMP-7 and the PFT indices, including %FVC and %DL_CO_ (ρ = −0.31, *p* = 0.02 and ρ = −0.32, p = 0.02, respectively). There were also significant correlations between MMP-10 and %FVC and %DL_CO_ (ρ = −0.34, *p* < 0.01 and *ρ* = −0.43, *p* < 0.01, respectively). There were significant correlations noted between MMP-10 and the 6-minute walk distance (*ρ* = −0.38, *p* < 0.01), as well as between MMP-10 and minimum oxygen saturation (SpO_2_) during the 6MWT (ρ = −0.42, *p* < 0.01). Only MMP-10 correlated significantly with the partial pressure of oxygen (ρ = −0.32, *p* = 0.02).

**Table 3:** Correlations between function measurements and serum matrix metalloproteinases in IPF^a^

	MMP-1	MMP-2	MMP-3	MMP-7	MMP-8	MMP-9	MMP-10	MMP-12
%FVC	–	–	–	-0.308^*^	–	–	-0.344^†^	–
%DL_CO_	–	–	-0.251	-0.316^*^	–	–	−0.434^†^	–
6MWD	–	−0.355^†^	–	–	−0.228	–	−0.384^†^	−0.309^*^
Minimum SpO_2_ during the 6MWT	–	–	−0.235	−0.261	–	–	−0.416^†^	–
PaO_2_ in room air	–	–	–	–	–	–	−0.318^*^	–

The data are shown as Spearman’s ρ when the *p*-value < 0.10

*IPF* idiopathic pulmonary fibrosis, *MMP* matrix metalloproteinase, *%FVC* percentage of predicted forced vital capacity, *%DL*_*CO*_ percentage of predicted diffusing capacity of the lung for carbon monoxide, *6MWD* 6-minute walk distance, *SpO*_*2*_ oxygen saturation, *6MWT* 6-minute walk test, *PaO*_*2*_ partial pressure of oxygen

^a^MMP-13 was excluded from the analysis

^*^ *p* < 0.05, ^†^ *p* < 0.01

### Correlation between serum and BALF MMPs

Based on the MMP results and their relationship with disease severity, the BALF concentrations of MMP-7 and -10 were measured. Among the 25 patients who underwent BAL, serum and BALF MMP measurements were simultaneously performed in 19 patients to investigate potential correlations. Significant correlations were observed between serum and BALF concentrations of MMP-7 and MMP-10 (ρ = 0.64, *p* < 0.01 and ρ = 0.47, *p* = 0.04, respectively). We also examined 25 BALF samples from the patients who underwent both BAL and PFT within a 1-month interval. BALF MMP-10 correlated significantly with %FVC and %DL_CO_ (ρ = −0.46, *p* = 0.02 and ρ = -0.44, *p* = 0.03, respectively), whereas BALF MMP-7 correlated significantly with only %DL_CO_ (ρ = −0.54, *p* = < 0.01).

### Relationship between MMPs and clinical deterioration or survival

The median follow-up time from baseline was 459 days (range, 12–1853 days). Fourteen patients deteriorated clinically within 6 months; 2 died due to an acute exacerbation (AE), and 9 exhibited declines in pulmonary function. Furthermore, 3 patients were admitted due to respiratory failure: 2 suffered from chronic respiratory failure, and 1 suffered from a respiratory infection. The causes of death at more than 6 months following baseline included 3 cases of AE, 2 cases of chronic respiratory failure, 1 case of acute lung injury due to pulmonary infection, 1 case of lung cancer, 1 case of sepsis, and 1 case without a clear diagnosis. Logistic analyses for clinical deterioration within 6 months revealed that serum MMP-10 was a significant predictor of clinical deterioration, as were %FVC and %DL_CO_ (Table 4A). A survival analysis performed using a Cox proportional hazard model demonstrated that serum MMP-10, as well as %FVC and %DL_CO_, was a significant predictor of mortality among patients with IPF (Table 4B). Serum MMP-10 was a significant predictor of clinical deterioration and mortality, even when the patients treated at baseline were excluded (Additional file 1: Table S2). In contrast, serum and BALF MMP-7 were not predictors of either clinical deterioration within 6 months or mortality.

**Table 4:** Univariate analyses utilized to predict clinical deterioration and mortality

(A) Logistic regression models utilized to predict clinical deterioration within 6 months			
	Odds ratio	95 % CI	*p*-value
%FVC	0.959	0.922–0.992	0.014
%DL_CO_	0.903	0.824–0.964	<0.001
Serum MMP-7			0.483
Serum MMP-10	2.716	1.184–7.830	0.017
BALF MMP-7			NA
BALF MMP-10			NA
(B) Cox hazard models utilized to predict mortality			
	Hazard ratio	95 % CI	*p*-value
%FVC	0.952	0.917–0.986	0.005
%DL_CO_	0.892	0.828–0.950	<0.001
Serum MMP-7			0.595
Serum MMP-10	1.773	1.032–2.887	0.039
BALF MMP-7			0.992
BALF MMP-10			0.377

The odds ratio and hazard ratio are shown when *p* < 0.10

*CI* confidence interval, *%FVC* percentage of predicted forced vital capacity, *%DL*_*CO*_ percentage of predicted diffusing capacity of the lung for carbon monoxide, *MMP* matrix metalloproteinase, *BALF* bronchoalveolar lavage fluid, *NA* not available

We performed an ROC analysis to determine the optimal cut-off value of serum MMP-10 for predicting clinical deterioration. The curve had an AUC of 0.741 and a cut-off value of 0.986 ng/μL. When 1.0 ng/μL was set as the threshold, patients with higher values had a significantly higher frequency of clinical deterioration within 6 months compared with patients with lowers values (*p* = 0.01). Furthermore, the mortality of the patients with higher serum MMP-10 values was significantly higher than patients with lower values (log rank test; *p* = 0.049).

### Immunohistochemistry

**Fig. 1:**
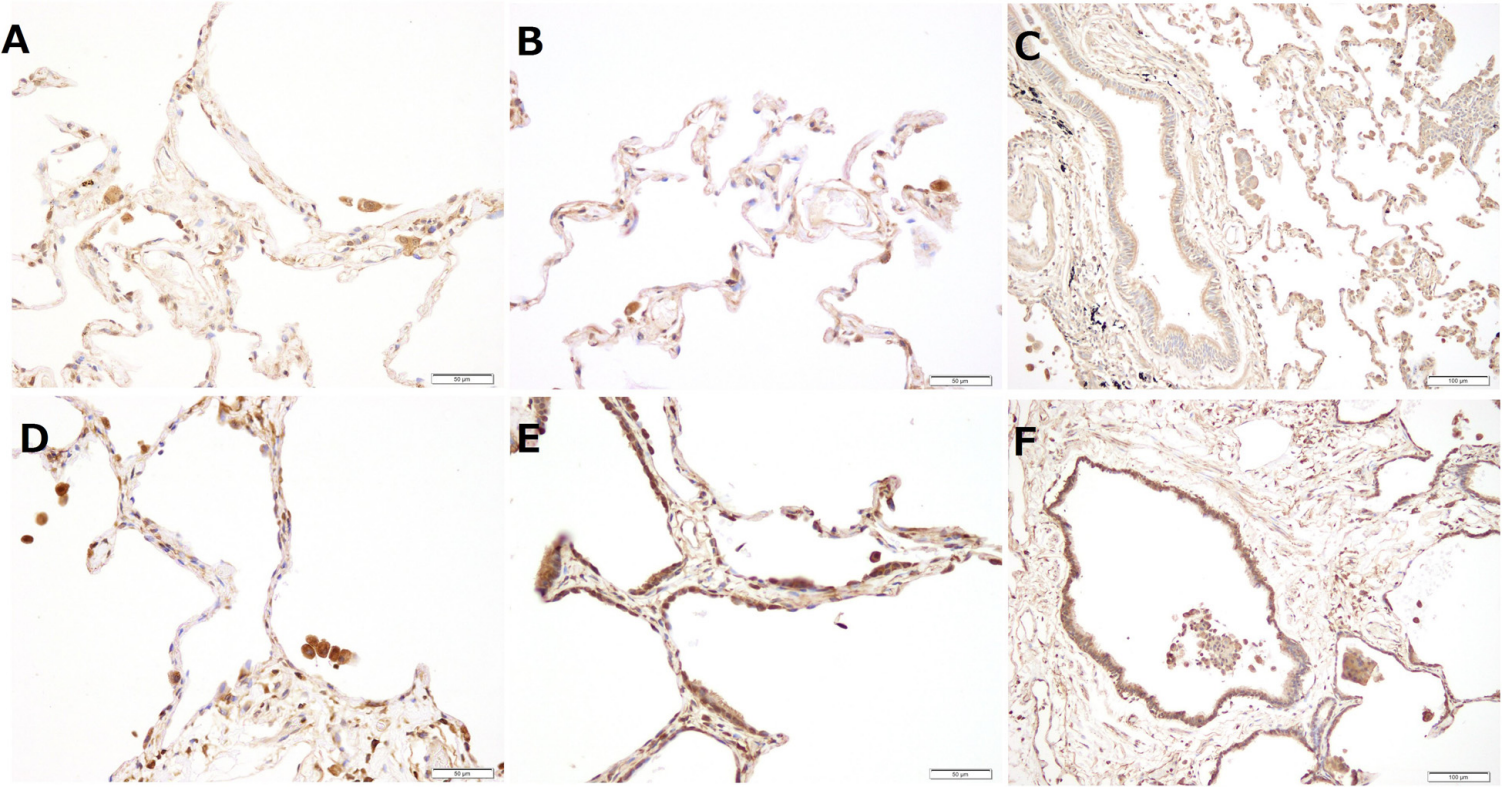
The immunohistochemical expression of matrix metalloproteinase (MMP)-10 in idiopathic pulmonary fibrosis (IPF) lung tissue and control lung tissue. (**a**-**c**) The expression of MMP-10 is weakly positive in alveolar epithelial cells and macrophages in control lung tissue. (**d**-**f**) In IPF lung tissue, MMP-10 is expressed predominantly in alveolar macrophages, alveolar epithelial cells, and peripheral bronchiolar epithelial cells. The staining intensity of MMP-10 in IPF lung tissue is stronger compared with control lung tissue

Immunohistochemical staining for MMP-10 was performed in IPF lung tissue. The expression of MMP-10 was localized primarily to the alveolar macrophages, alveolar epithelial cells, and peripheral bronchiolar epithelial cells in IPF lung tissue (Fig. 1). Positive immunostaining for MMP-10 was observed in both macrophages and alveolar epithelial cells in control lung tissue. However, the number of positive cells was reduced, and the signal intensity was weaker in control lung tissue compared with IPF lung tissue.

## Discussion

In the present study, several MMPs, including MMP-10, were significantly elevated in the sera of the patients with IPF compared with the patients with COPD and controls. MMP-10 and -7 correlated significantly with several physiological indices, including %FVC and %DL_CO_. Furthermore, a higher serum MMP-10 level was associated with both clinical deterioration within 6 months and overall survival. In IPF lungs, MMP-10 was expressed in alveolar macrophages and epithelial cells.

MMPs reportedly play an important role in the pathogenesis of IPF [13]. Previous studies have demonstrated that both BALF and serum MMP-1 were elevated in IPF [14, 15]. The biological roles of MMP-1 suggest that it may play an important role in the pathogenesis of IPF [13, 24]. MMP-3-knockout mice and MMP-7-knockout mice do not develop pulmonary fibrosis induced by bleomycin [25, 26], suggesting that MMP-3 and -7 may also act as mediators of pulmonary fibrosis. Indeed, MMP-1 and -7 were also increased in the patients with IPF compared with controls in the present study.

In previous studies, however, few MMPs reportedly correlated with disease severity and prognosis despite their possible roles in the pathogenesis of IPF. Serum MMP-7 concentrations were elevated in patients with subclinical interstitial lung disease (ILD) and correlated negatively with %FVC and %DL_CO_, whereas no significant correlation was noted between serum MMP-1 and pulmonary function [15]. High levels serum MMP-7 and SP-A may predict shorter survival in patients with IPF [27]. In the present study, we demonstrated a correlation between MMP-7 and pulmonary function, including %FVC and %DL_CO_, that was consistent with the findings of the previous studies. Furthermore, we observed that serum MMP-10 correlated more significantly with disease severity and prognosis in patients with IPF compared with MMP-7.

MMP-10, like MMP-3 and -11, is categorized into stromelysins and degrades a variety of ECM proteins, including proteoglycans, laminin, fibronectin, gelatins, and collagen types III, IV, V, and IX [18, 28, 29]. MMP-10 activates other MMPs, including proMMP-1, -7, -8, and -9 [17, 30]. MMP-10 expression is observed in injured and remodeling tissues as well as in various types of cells, including the keratinocytes present in skin wounds, injured colonic tissue, and different cells in injured liver [18, 31, 32]. MMP-10 is one of the most well-known MMPs involved in the pathogenesis of carcinomas. Elevated levels of MMP-10 protein have been observed in tumors, including non-small cell lung cancer [33], head and neck squamous cell carcinoma [34], bladder transitional cell carcinoma [35], epithelial skin cancer [36], renal cell carcinoma [37], and colon adenocarcinoma [38].

In experimental models, MMP-10 was elevated via exposure to cerium oxide or silica [39, 40]. A previous study evaluating the degree of pulmonary fibrosis induced by ultrafine amorphous silica also demonstrated that the expression of MMP-10 was associated with pulmonary fibrosis [41]. Transforming growth factor-β (TGF-β) is elevated in IPF and exerts profibrotic effects, such as fibroblast differentiation, suppression of myofibroblast apoptosis, ECM induction, and regulation of the balance between MMPs and tissue inhibitors of MMPs [42, 43]. Furthermore, TGF-β up-regulates several MMPs, including MMP-10, in epithelial cells [44, 45]. Therefore, pulmonary fibrosis may be induced by TGF-β through several pathways, including MMP-10. Taken together, MMP-10 may be involved in the pathogenesis of pulmonary fibrosis, as with other MMPs, such as MMP-1 and -7.

We observed that in human fibrotic lungs, the expression of MMP-10 was localized to the alveolar epithelial cells, macrophages, and peripheral bronchiolar epithelial cells. MMP-10 reportedly localized to fibrotic regions and alveolar macrophages in cerium oxide-treated lungs and silica-induced pulmonary fibrosis [39, 40]. However, the localization of MMP-10 has not been investigated in IPF. Most MMPs, including MMP-1, -2, -7, and -9, are expressed in alveolar epithelial cells, whereas other MMPs, including MMP-2 and -9, may be found in the fibroblastic foci in IPF [2]. Immunohistochemical results and the significant correlation between serum and BALF MMP-10 observed in the present study suggest that serum MMP-10 in IPF is derived from the epithelial cells and macrophages in the lungs.

Despite a more significant correlation of serum MMP-10 with disease severity and prognosis compared with other MMPs, serum concentrations of MMP-10 were lower than many other MMPs in IPF. Although the reason was unclear, the abundant MMP-10 expression in IPF lungs visualized by immunological staining and the significant association between serum MMP-10 and clinical features may suggest that MMP-10 plays a significant role in the pathogenesis of IPF.

There were some limitations to the present study. First, the number of patients was small. Additional prospective studies with larger numbers of patients are necessary to validate the role of MMP-10 as a biomarker for IPF. Second, we did not investigate MMPs in other ILDs, such as nonspecific interstitial pneumonia, connective tissue disease-associated ILD, and OP. MMP-7 is reportedly elevated in other ILDs, such as cryptogenic OP and systemic sclerosis-associated ILD [46–48]; therefore, the up-regulation of MMP-10 may not be specific to IPF. Third, some patients had already been treated with prednisolone, an immunosuppressant, or pirfenidone at baseline. Pirfenidone, an antifibrotic agent that suppresses TGF-β, may affect the expression of MMPs, including MMP-10 [49]. However, the prognostic significance of serum MMP-10 persisted after excluding the treated patients.

We concluded that MMP-10 is a novel biomarker for IPF, correlates with disease severity, and predicts disease progression. Additional studies are necessary to elucidate the functional role of MMP-10 and other MMPs in the pathogenesis of IPF.

## Additional file

Additional file 1: Table S1. Treatment for IPF at baseline. **Table S2.** Univariate analyses utilized to predict clinical deterioration and mortality when 13 treated patients with IPF were excluded. (DOCX 24 kb)

## Abbreviations

IPF: idiopathic pulmonary fibrosis
FVC: forced vital capacity
DL_CO_: diffusing capacity of the lung for carbon monoxide
SP-A: surfactant protein-A
MMP: matrix metalloproteinase
ECM: extracellular matrix
BALF: bronchoalveolar lavage fluid
FEV_1_: forced expiratory volume in one second
COPD: chronic obstructive pulmonary disease
GOLD: Global Initiative for Chronic Obstructive Lung Disease
PFT: pulmonary function test
6MWT: 6-minute walk test
ABG: arterial blood gas
BAL: bronchoalveolar lavage
%FVC: percentage of predicted FVC
%DL_CO_: percentage of predicted DL_CO_
OP: organizing pneumonia
ROC: receiver operating characteristic
UIP: usual interstitial pneumonia
SpO_2_: oxygen saturation
AE: acute exacerbation
ILD: interstitial lung disease
TGF-β: transforming growth factor-β
