# Supplementary material for: Matrix metalloproteinase-10: a novel biomarker for idiopathic pulmonary fibrosis
Source: Respir Res. 2015 Sep 29;16:120. doi: 10.1186/s12931-015-0280-9 (PMC4587921; doi:10.1186/s12931-015-0280-9)
Supplement: Additional file 1: Table S1. — Treatment for IPF at baseline. Table S2. Univariate analyses utilized to predict clinical deterioration and mortality when 13 treated patients with IPF were excluded. (DOCX 24 kb) [file 12931_2015_280_MOESM1_ESM.docx]

**Additional file 1**

**Table S1.** Treatment for IPF at baseline

| Treatment |  |
| --- | --- |
| None | 44 (77.2) |
| Only corticosteroids | 2 (3.5) |
| Only pirfenidone | 7 (12.3) |
| Corticosteroids + IS | 3 (5.3) |
| Corticosteroids + pirfenidone | 1 (1.7) |

The data are presented as the number (%)

IPF = idiopathic pulmonary fibrosis; IS = immunosuppressant.

**Table S2.** Univariate analyses utilized to predict clinical deterioration and mortality when 13 treated patients with IPF were excluded.

(A) Logistic regression models utilized to predict clinical deterioration within 6 months

|  | Odds ratio | 95% CI | p-value |
| --- | --- | --- | --- |
| %FVC |  |  | 0.329 |
| %DL_CO_ | 0.936 | 0.850 – 1.005 | 0.071 |
| Serum MMP-7 |  |  | 0.708 |
| Serum MMP-10 | 2.606 | 1.076 – 8.244 | 0.034 |
| BALF MMP-7 |  |  | NA |
| BALF MMP-10 |  |  | NA |

(B) Cox hazard models utilized to predict mortality

|  | Hazard ratio | 95% CI | p-value |
| --- | --- | --- | --- |
| %FVC | 0.950 | 0.906 – 0.994 | 0.027 |
| %DL_CO_ | 0.901 | 0.807 – 0.980 | 0.012 |
| Serum MMP-7 |  |  | 0.736 |
| Serum MMP-10 | 1.895 | 1.007 – 3.436 | 0.048 |
| BALF MMP-7 |  |  | 0.992 |
| BALF MMP-10 |  |  | 0.377 |

The odds ratio and hazard ratio are shown when p < 0.10.

CI = confidence interval; %FVC = percentage of predicted forced vital capacity; %DL_CO_ = percentage of predicted diffusing capacity of the lung for carbon monoxide; MMP = matrix metalloproteinase; BALF = bronchoalveolar lavage fluid; NA = not available.
